# Patients with chronic ankle instability exhibit increased sensorimotor cortex activation and correlation with poorer lateral balance control ability during single-leg stance: a FNIRS study

**DOI:** 10.3389/fnhum.2024.1366443

**Published:** 2024-04-26

**Authors:** Na Liu, Chen Yang, Qipeng Song, Fengying Yang, Yan Chen

**Affiliations:** College of Sport and Health, Shandong Sport University, Jinan, Shandong, China

**Keywords:** chronic ankle instability, single-leg stance, balance, sensorimotor cortex, functional near-infrared spectroscopy

## Abstract

**Introduction:**

Chronic Ankle Instability (CAI) is a musculoskeletal condition that evolves from acute ankle sprains, and its underlying mechanisms have yet to reach a consensus. Mounting evidence suggests that neuroplastic changes in the brain following ankle injuries play a pivotal role in the development of CAI. Balance deficits are a significant risk factor associated with CAI, yet there is a scarcity of evidence regarding the sensorimotor cortical plasticity related to balance control in affected individuals. This study aims to evaluate the differences in cortical activity and balance abilities between patients with CAI and uninjured individuals during a single-leg stance, as well as the correlation between these factors, in order to elucidate the neurophysiological alterations in balance control among patients with CAI.

**Methods:**

The study enrolled 24 patients with CAI and 24 uninjured participants. During single-leg stance, cortical activity was measured using a functional near-infrared spectroscopy (fNIRS) system, which included assessments of the pre-motor cortex (PMC), supplementary motor area (SMA), primary motor cortex (M1), and primary somatosensory cortex (S1). Concurrently, balance parameters were tested utilizing a three-dimensional force platform.

**Results:**

Independent sample *t-*tests revealed that, compared with the uninjured individuals, the patients with CAI exhibited a significant increase in the changes of oxyhemoglobin concentration (ΔHbO) during single-leg stance within the left S1 at Channel 5 (*t* = 2.101, *p* = 0.041, Cohen’s *d* = 0.607), left M1 at Channel 6 (*t* = 2.363, *p* = 0.022, Cohen’s *d* = 0.682), right M1 at Channel 15 (*t* = 2.273, *p* = 0.029, Cohen’s *d* = 0.656), and right PMC/SMA at Channel 11 (*t* = 2.467, *p* = 0.018, Cohen’s *d* = 0.712). Additionally, the center of pressure root mean square (COP-RMS) in the mediolateral (ML) direction was significantly greater (*t* = 2.630, *p* = 0.012, Cohen’s *d* = 0.759) in the patients with CAI. Furthermore, a moderate positive correlation was found between ML direction COP-RMS and ΔHbO2 in the M1 (*r* = 0.436; *p* = 0.033) and PMC/SMA (*r* = 0.488, *p* = 0.016), as well as between anteroposterior (AP) direction COP-RMS and ΔHbO in the M1 (*r* = 0.483, *p* = 0.017).

**Conclusion:**

Patients with CAI demonstrate increased cortical activation in the bilateral M1, ipsilateral PMC/SMA, and contralateral S1. This suggests that patients with CAI may require additional brain resources to maintain balance during single-leg stance, representing a compensatory mechanism to uphold task performance amidst diminished lateral balance ability in the ankle joint.

## 1 Introduction

Ankle sprains are among the most common sport-related injuries encountered in competitive athletics, military training, and everyday life activities (Doherty et al., 2014), accounting for approximately 10–30% of all sports injuries (Herzog et al., 2019). 70% of the overall population experience at least one ankle sprain in their lifetime, with 85% of these being inversion sprains that compromise the structural integrity of the lateral ligaments of the ankle (Hiller et al., 2012). A history of at least one previous ankle sprain is the primary risk factor for high recurrence rate (Wikstrom et al., 2013) with approximately 30–78% in common people and above 80% in athletes (Gribble et al., 2013). Ankle sprains result in an average loss of 7–29 days of productivity and an average medical cost of $1,029 (Wagemans et al., 2023), cumulating to an annual expense of $3.8 billion (Gerber et al., 1998). Within the first year following an initial ankle sprain, 40% of patients develop chronic ankle instability (CAI), characterized by recurrent sprains, pain, swelling, weakness, a sensation of instability, and functional impairment, leading to 20–40% of affected individuals ceasing sports activities (Zhang et al., 2022), even persistent disability for more than 7 years and increased risk of developing post-traumatic ankle osteoarthritis (Terada et al., 2015).

Despite extensive investigation into the pathomechanics of CAI over the past 60 years, consensus has not been reached (Wikstrom and Brown, 2014; Needle et al., 2017; Hertel and Corbett, 2019). Most traditional models consider CAI to be a musculoskeletal disorder, with adaptations regularly occurring in the periphery rather than in the brain (Needle et al., 2017). However, increasing evidence supports the notion that alterations in ankle movement post-injury may lead to adaptations in the central nervous system (CNS) (Kim et al., 2019), specially associated with reorganization of the cerebral cortex (Nanbancha et al., 2019). Neuroplasticity refers to the brain’s ability to reorganize synaptic connections, functional networks, or morphological structures responding to stimuli such as peripheral injuries (Shen et al., 2022). Insufficient ligament afferent, proprioceptive, and other somatosensory inputs may induce maladaptive sensorimotor functional adaptations, limiting the brain to regulate movement and joint stability via descending neuromuscular pathways (Nyland et al., 2017), where patients with CAI manifest self-reported functional impairments and the recovery process interference (Pietrosimone and Gribble, 2012; Houston et al., 2015). A latest study suggests that compared to the CAI and uninjured groups, coper group (individuals who have experienced an ankle sprain, fully recovered, and did not develop CAI) exhibited greater activation in the primary somatosensory cortex (S1) and the superior temporal gyrus (STG) during single-leg stance, while no difference was observed between the CAI and uninjured groups (Ma et al., 2023).

Poor stability of the ankle joint, closest to the supporting base of the body, leads to decreased balance ability with the resultant increase of the ankle injury risk by fourfold (Burcal et al., 2019). The Single-Leg Stance (SLS), the integral component of various activities in daily living (Kurz et al., 2018), serves as a good indicator for assessing the balance ability (Ema et al., 2016). Poor SLS is regarded as a risk factor for recurrent injuries and CAI (Kim, 2020). Balance control is a complex motor task reliant on the integration of multimodal information within the sensorimotor cortical network (Sherman et al., 2021). Balance control deficits in patients with CAI may be associated with functional and structural changes in brain regions related to sensorimotor processing (Maricot et al., 2023). Neuroimaging studies indicate that direct motor networks, such as the primary motor cortex (M1) and cerebellum, as well as indirect motor networks, including the prefrontal cortex (PFC) and supplementary motor area (SMA), are responsible for balance control (Herold et al., 2017). Limited evidence suggests that cortical activation is associated with lower limb movement, postural control, and balance ability (Linortner et al., 2014), due to the limited availability of advanced technologies for direct assessment of brain activity during balance tasks.

Functional near-infrared spectroscopy (fNIRS) is an optical neuroimaging technology based on the theory of neurovascular coupling, which detects differences in the absorption spectra of oxyhemoglobin (HbO) and deoxyhemoglobin (HbR) within the near-infrared spectrum, thereby indirectly assessing cortical activation (Sakudo, 2016). The fNIRS technology offers high temporal resolution and non-invasively measures task-related cortical responses, proving to be safer and more reliable (Jöbsis, 1977). Importantly, due to its robustness to subject movement, fNIRS is an ideal tool for studying cortical activity in postural balance control (Mihara et al., 2008).

To date, various treatment strategies for CAI, including external support, exercise therapy, and stabilization surgery, have been employed to restore ankle stability. Yet, some patients still face residual symptoms and the inability to return to sports (Doherty et al., 2017; Song et al., 2019), indicating that current rehabilitation measures may be insufficient to reduce the rates of recurrent sprains and CAI. Therefore, exploring the neural mechanisms behind CAI can enhance our understanding of the condition and aid in the development of appropriate neurorehabilitation strategies. To this end, we assessed cortical activation with fNIRS and balance ability with a three-dimensional force platform in patients with CAI during SLS, as well as the relationship between the two. We hypothesized that compared to uninjured individuals, patients with CAI exhibited higher levels of cortical activation, poorer balance ability, and a correlation between them during the SLS task.

## 2 Participants and methods

### 2.1 Participants

This study utilized a matched group design. Sample size calculations were performed using G*Power 3.1.9 software, taking the variability of supplementary motor area (SMA) cortical activation in patients with CAI from a relevant study to obtain an effect size (Cohen’s *d*) of 0.82 (Rosen et al., 2019). A sample size of 20 participants per group (totaling 40 participants) was estimated with alpha level of 0.05 and statistical power of 0.80. Participants were recruited from the campus of Shandong Sport University, where their ankle function and injury history were assessed. A total of 48 subjects were selected to participate in this study, including 24 individuals with unilateral chronic ankle instability (CAI group) and 24 individuals without a history of ankle injuries (Control group). The CAI group included patients with injuries on both dominant and non-dominant sides. The gender, age, height, and weight of the uninjured participants were matched with those of the CAI group. The lower limb tested was determined by referencing the injured limb (dominant or non-dominant side) of the patients with CAI. Limb dominance was defined as the leg that participants reported preferring to kick a ball or draw the Figure 8 on the floor (Li et al., 2018).

Following the recommendations of the International Ankle Consortium (Gribble et al., 2014), the inclusion criteria for the patients with CAI were: (1) a history of at least one significant ankle sprain occurring at least 12 months prior to the experiment, which resulted in pain and swelling, and at least one interruption of desired physical activities for a day; (2) the previously injured ankle having experienced at least two instances of “giving way” and/or recurrent sprains and/or “feelings of instability” within 6 months prior to study enrollment, with the most recent ankle sprain occurring at least 3 months before participation in the study; (3) a score of ≤24 on the Cumberland Ankle Instability Tool (CAIT), a score of ≥11 on the Identification of Functional Ankle Instability (IdFAI) questionnaire, and scores of <90% on the Foot and Ankle Ability Measure for the activities of daily living (FAAM-ADL) subscale and < 80% for the sports (FAAM-S) subscale. The inclusion criteria for the uninjured participants were: (1) no history of sprains in either ankle; (2) absence of instability or “giving way” in both ankles; (3) a score of ≥28 on the CAIT, a score of 0 on the IdFAI, and 100% scores on the FAAM-ADL and FAAM-S subscales.

The exclusion criteria for all participants were (Gribble et al., 2014): (1) a history of fractures or surgery on any of the lower limbs; (2) an acute musculoskeletal injury to other joints of the lower limbs in the past 3 months, which affected joint integrity and function, resulting in at least one interruption of desired physical activities for a day; (3) a diagnosed balance or vestibular disorder; (4) confirmed neurological damage or psychiatric illness.

All participants were requested to refrain from consuming any caffeine, alcohol, or stimulants 24 h prior to the experiment. Before the experiments commenced, the purpose and the procedures were explained to all participants, and written informed consent was obtained. The experimental study was approved by the Ethics Committee of Shandong Sport University (Approval Number: 2022023), and all methods were conducted in accordance with the latest guidelines and regulations of the Declaration of Helsinki.

### 2.2 Experimental procedure

**Figure 1:**
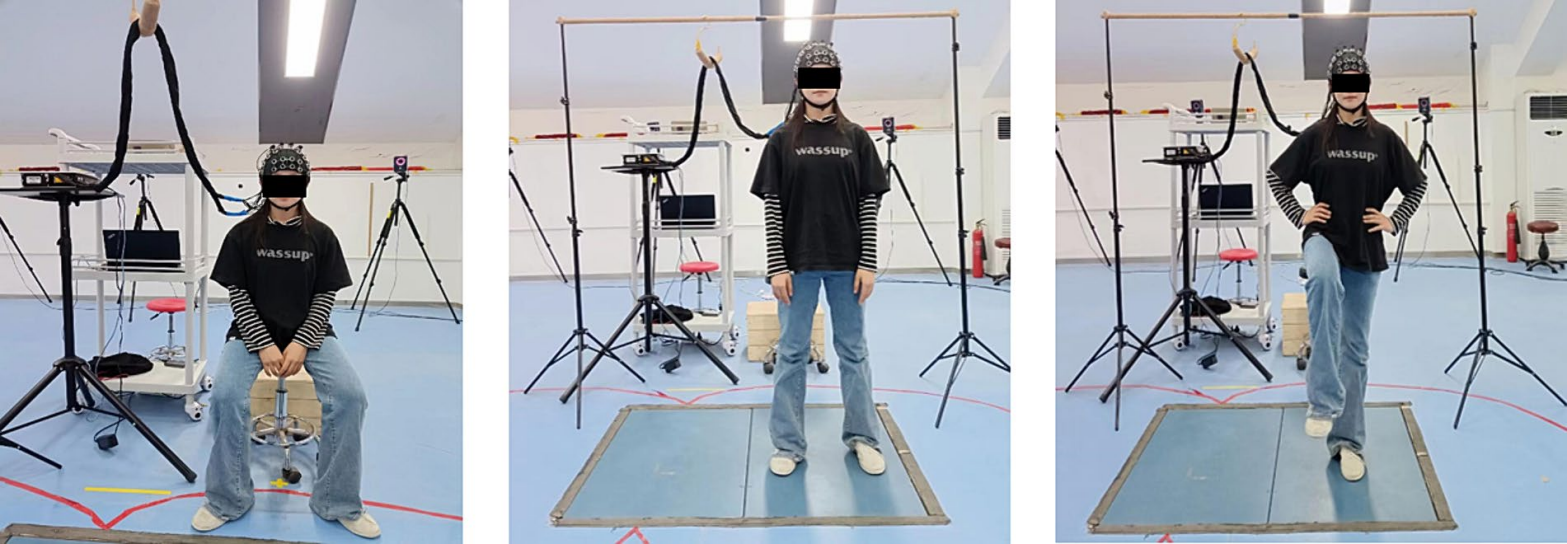
A flow chart of the testing process.

The experiment was conducted at the Sport Biomechanics Laboratory of Shandong Sport University. It consisted of three trials and each trial included 30 s of quiet sitting (baseline) followed by 30 s of single-leg stance (task). Initially, to obtain the baseline of non-task-related brain activity for data analysis, the participants were asked to remain in quiet sitting for 30 s. Subsequently, the participants stood on a force platform with their arms naturally placed at their sides and their feet parted shoulder-width apart. The researchers initiated a cue sound “Start” to simultaneously begin collecting data on cerebral hemodynamics and plantar pressure. The participants were instructed to use their affected leg as the support limb, with hands placed on the iliac crests and the contralateral limb held with approximately 45 degrees of knee flexion and 30 degrees of hip flexion (Hertel et al., 2001). They maintained a stable head posture, focusing attention on a visual target at eye level on the wall in front of them to minimize eye movements. The test was concluded upon hearing the cue sound “Stop” (see Figure 1). There was a minimum rest period of 1 min between trials. Each participant performed only one task trial prior to the actual test procedure to minimize any practice or learning effects (Elsotohy et al., 2021). The experiment was performed in a quiet environment because an external focus can affect cortical activity during single-leg stance (Sherman et al., 2021).

To minimize potential confounding effects, the participants were instructed to limit any unnecessary movements such as clenching teeth or facial expressions during the testing process. If these events occurred, for example, non-supportive foot contacting with the force platform, contacting between the non-supportive and supportive foot, movement or hopping of the supportive foot, lifting of the forefoot or heel, hands leaving the waist, and hip abduction or lateral bending of the torso beyond 30 degrees, the data were invalidated. The participant had a break, and the trial was repeated until three valid trials were completed.

### 2.3 Data acquisition

#### 2.3.1 Hemodynamics

**Figure 2:**
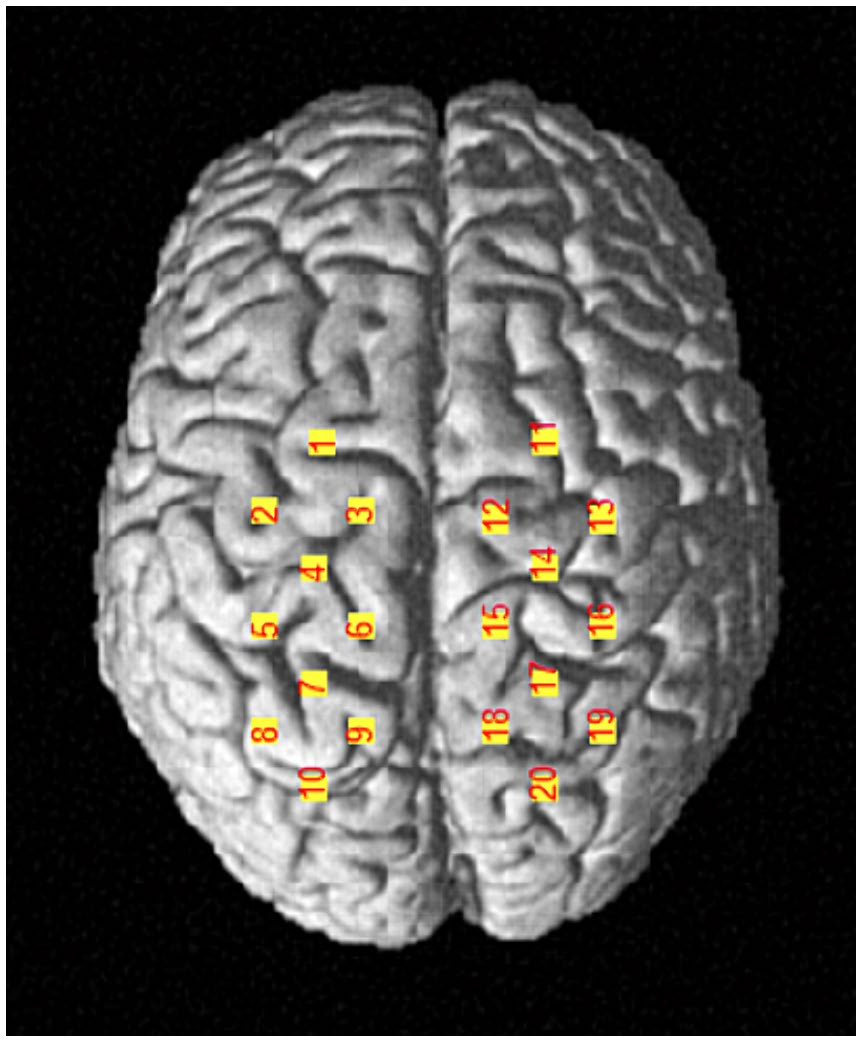
A diagram of the channel positions.

A portable near-infrared brain imaging system (LIGHTNIRS, Shimadzu Corp., Kyoto, Japan) was utilized to record the hemodynamic signals of localized brain regions during the single-leg stance task. The device included 8 emitter and 8 detector optodes, crosswise arranged in a 4 × 4 array with 20 channels. The distance between adjacent optodes was 3 cm. The wavelengths were 780, 805, and 830 nm, with a sampling frequency of 13.3 Hz. According to the international 10–20 system for EEG, the Cz was located at the intersection of the line between the preauricular points and the line from the nasion to the inion. Channels 3 and 12 were placed to the left and right of Cz, respectively. This configuration allows for the detection of the primary motor cortex (M1), pre-motor cortex (PMC), supplementary motor area (SMA), and primary somatosensory cortex (S1). Each optode was checked, and the hair beneath it was parted to ensure that the optode probes were in full contact with the scalp. The optodes were covered with an opaque black cloth to reduce interference from external light. A three-dimensional (3D) digitizer (FASTRAK, Polhemus, Vermont) was used to determine the optode and channel Montreal Neurological Institute (MNI) coordinates Supplementary Table 1, as well as the corresponding brain locations for each channel (Okamoto et al., 2004; Figure 2). The left M1 was covered by channels 4 and 6, while the right M1 was covered by channels 14 and 15; the left PMC/SMA by channels 1, 2, and 3, and the right PMC/SMA by channels 11, 12, and 13; the left S1 by channel 5, and the right S1 by channel 16.

#### 2.3.2 Center of pressure

A three-dimensional force platform (AMTI, Inc., Watertown, MA, United States) equipped with Vicon Nexus 1.7.1 software was used to collect the sway trajectory of the center of pressure (COP) during single-leg stance (sampling frequency 1,000 Hz). The force platform is widely utilized for quantitative postural balance assessment in laboratory settings (Piirtola and Era, 2006). The COP is considered the gold standard for evaluating balance abilities (Huurnink et al., 2013), with parameters derived from COP trajectories used to indicate balance deficits (i.e., larger COP sway signifies poorer balance control) (Xue et al., 2023).

### 2.4 Data processing

#### 2.4.1 Hemodynamics

Data preprocessing was conducted using the Homer2 toolbox, which is based on MATLAB (R2013b, MathWorks Inc., Natick, United States) (Huppert et al., 2009). The differences between each task and the baseline were calculated to determine the cerebral hemodynamic changes induced by single-leg stance. The raw data were first visually inspected to mark the bad channels and to remove the segments with poor signal quality. The optical intensity data were converted into optical density data. Artifact correction was performed using linear interpolation. Subsequently, the signal was band-pass filtered with a high-pass cut-off frequency (0.01 Hz) to eliminate instrumental noise and low-frequency drift, and a low-pass cut-off frequency (0.1 Hz) to remove the spontaneously generated physiological components (Mayer waves: ≈0.1 Hz, heart rate: 1.6–1.8 Hz, respiration: 0.2–0.3 Hz) (Pinti et al., 2018). Using the modified Beer–Lambert Law, optical density data were converted to the concentrations of oxyhemoglobin (HbO) and deoxyhemoglobin (HbR) (Brigadoi et al., 2014). The mean value of HbO within the last 5 s of each sitting was selected as a baseline for correction, resulting in the oxyhemoglobin concentration changes (ΔHbO). The ΔHbO for all sampling points during each task and across the three trials were then averaged for statistical analysis. The HbO data were chosen, because HbO signals have a better signal-to-noise ratio (Kumar et al., 2017), higher reproducibility (Zhang et al., 2011), are more sensitive indicators of local blood flow changes (Hoshi, 2003)and have been proven to be superior in assessing functional activity. The BrainNet Viewer toolkit based on MATLAB was used for the visualization of cortical activations (Xia et al., 2013).

#### 2.4.2 Center of pressure

During the stance phase for 30 s, the ground reaction force data were collected and filtered using a fourth-order low-pass Butterworth filter (cut-off frequency 12 Hz). The displacement of the COP trajectory in the anteroposterior (AP) and mediolateral (ML) directions was calculated. COP relevant indices are frequently used to assess balance control during single-leg stance (van Dieën et al., 2015). The COP root mean square (COP-RMS) is a time-domain measurement that represents the average variance of the signal captured during the balance test (Neville et al., 2015) and has been used by many studies as an indicator of static balance control. The filtered data were used to calculate the RMS of COP displacement for each participant in the AP (COP-RMSap) and ML (COP-RMSml) directions. A higher value indicates poorer balance control ability in either direction. The calculation formulas are as follows:


$$\mathrm{COP}-\mathrm{RMSap}=\sqrt{\frac{\sum {\left(x_{i}-x\right)}^{2}}{N-1}};\;\mathrm{COP}-\mathrm{RMSml}=\sqrt{\frac{\sum {\left(y_{i}-y\right)}^{2}}{N-1.}}$$

In these equations, x and y represent the average position of the COP in the AP and ML directions.

### 2.5 Statistical analysis

Statistical analysis was performed using IBM SPSS Statistics 26.0. The Shapiro–Wilk Test was employed to assess the normality of the data. If the data were normally distributed, independent sample T-test was used to compare the differences in cortical ΔHbO and plantar COP-RMS between the CAI group and the Control group. The Cohen’s d was utilized to quantitatively assess the effect size, with the effect size categorized as follows: 0.0–0.2, trivial; 0.2–0.6, moderate; 0.6–1.2, large; >1.2, very large (Bernards et al., 2017). To investigate potential links between cortical activity and balance parameters, Pearson correlation analysis was conducted. The thresholds for the correlation coefficient (*r*) were as follows: weak (0–0.4), moderate (0.4–0.7), or strong (0.7–1.0) (Pietrosimone and Gribble, 2012). The data were presented as mean ± standard error (M ± SEM). The level of significance was set at *p* < 0.05.

## 3 Results

### 3.1 Demographic data

Table 1 summarizes the demographic data of the participants in both groups. There were no significant differences between the two groups in terms of gender, age, height, weight and dominant/non-dominant leg (*p* > 0.05).

**Table 1:** The demographic data of the participants.

Characteristics	CAI group (*n* = 24)	Control group (*n* = 24)	t	P
Gender (male/female)	12/12	12/12		
Dominant/non-dominant leg	19/5	19/5		
Age (year)	21.54 ± 0.37	21.92 ± 0.33	−0.756	0.453
Height (cm)	172.00 ± 2.08	172.71 ± 1.78	−0.259	0.797
Weight (kg)	66.13 ± 2.59	67.42 ± 2.51	−0.358	0.722

### 3.2 Hemodynamics data

**Figure 3:**
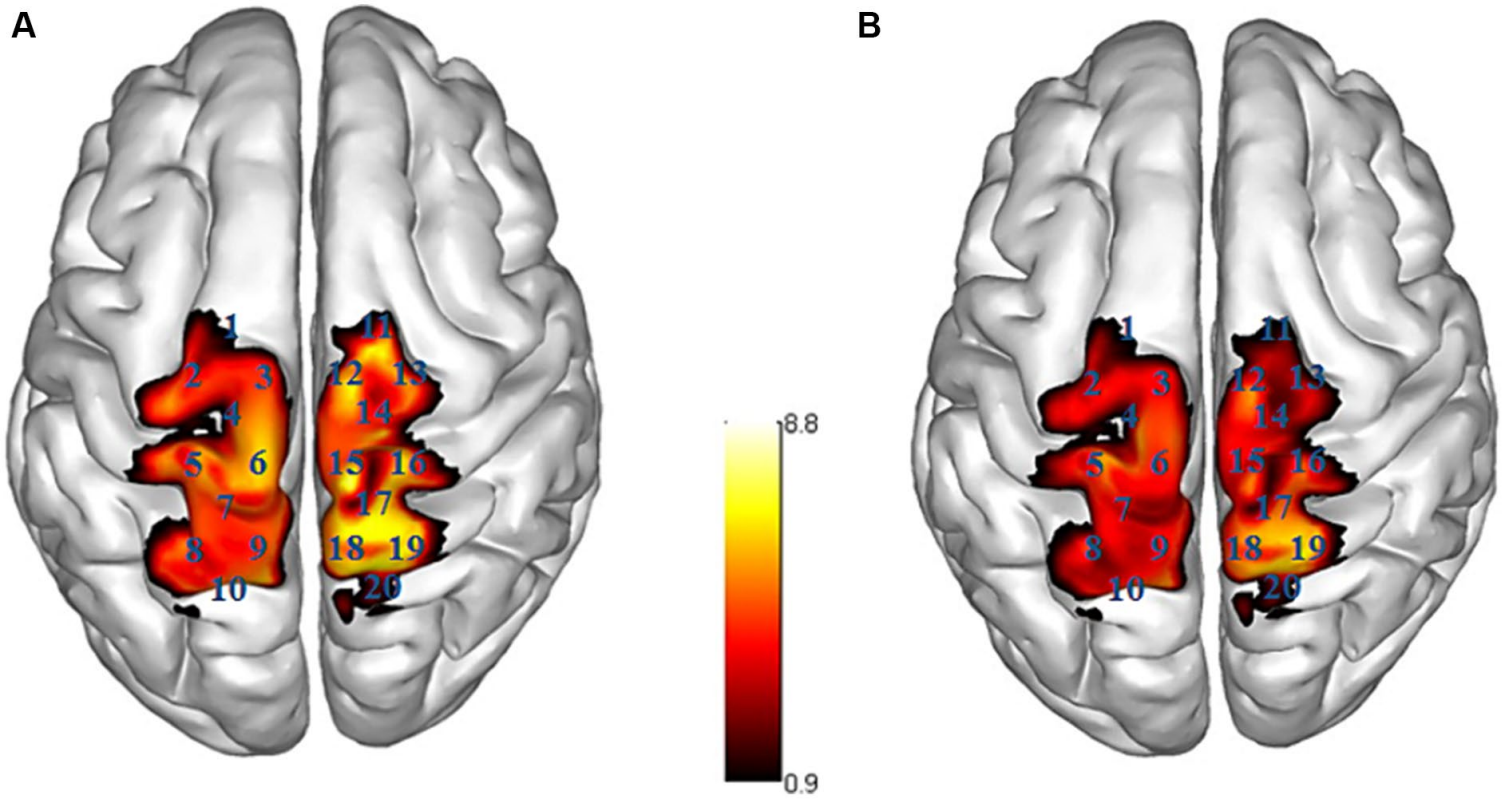
Activation map of brain areas during single-leg stance. R right side, L left side; **(A)** CAI group, **(B)** Control group; the ΔHbO value is larger, the color is brighter.

The ΔHbO of the participants in the two groups revealed significant differences in Channel 5 (left S1, *t* = 2.101, *p* = 0.041, Cohen’s *d* = 0.607), Channel 6 (left M1, *t* = 2.363, *p* = 0.022, Cohen’s *d* = 0.682), Channel 15 (right M1, *t* = 2.273, *p* = 0.029, Cohen’s *d* = 0.656), and Channel 11 (right PMC/SMA, *t* = 2.467, *p* = 0.018, Cohen’s *d* = 0.712), with the CAI group showing higher ΔHbO compared to the Control group. No significant differences were found in the remaining channels (see Table 2 and Figure 3).

**Table 2:** The ΔHbO of 20 channels in CAI group and Control group (× 10^−7^ mmol/L).

Brain area	Channel	CAI group	Control group	t	P
Premotor cortex/supplementary motor area (PMC/SMA)	1	3.484 ± 1.863	1.479 ± 1.993	0.735	0.466
	2	9.678 ± 2.274	4.877 ± 2.823	1.324	0.192
	3	4.384 ± 1.747	1.883 ± 2.034	0.933	0.356
	11	8.155 ± 2.360^*^	1.145 ± 1.583	2.467	0.018
	12	8.769 ± 2.179	4.642 ± 1.856	1.442	0.156
	13	6.766 ± 2.561	2.611 ± 1.870	1.310	0.197
Primary motor cortex (M1)	4	6.549 ± 2.290	4.110 ± 2.359	0.742	0.462
	6	6.396 ± 1.352^*^	1.388 ± 1.632	2.363	0.022
	14	3.934 ± 2.195	1.280 ± 1.615	0.974	0.335
	15	8.524 ± 2.154^*^	3.016 ± 1.109	2.273	0.029
Primary somatosensory cortex (S1)	5	7.550 ± 2.094^*^	1.130 ± 2.225	2.101	0.041
	16	6.162 ± 2.033	2.845 ± 1.889	1.195	0.238
Somatosensory Association Cortex (SAC)	7	5.337 ± 1.699	1.438 ± 1.870	1.543	0.130
	8	4.433 ± 2.521	0.959 ± 1.879	1.105	0.275
	9	3.933 ± 1.933	1.456 ± 1.444	1.027	0.310
	10	6.883 ± 1.819	3.823 ± 1.808	1.193	0.239
	17	7.216 ± 1.890	3.684 ± 2.172	1.226	0.226
	18	7.575 ± 2.183	4.190 ± 2.286	1.071	0.290
	19	8.516 ± 2.030	5.277 ± 1.856	1.178	0.245
	20	8.317 ± 2.294	3.735 ± 2.678	1.300	0.200

*Represents significant difference, *P* < 0.05.

### 3.3 Center of pressure data

The COP-RMSap showed no significant difference between the CAI group and the Control group. However, the COP-RMSml in the CAI group was significantly larger than the Control group (*t* = 2.630, *p* = 0.012, Cohen’s *d* = 0.759) (see Table 3).

**Table 3:** COP-RMS during single-leg stance in CAI group and Control group (mm).

Indicator	CAI group	Control group	t	p
COP-RMSap	6.641 ± 0.158	6.535 ± 0.238	0.371	0.713
COP-RMSml	9.307 ± 0.333^*^	7.987 ± 0.375	2.630	0.012

*Represents significant difference, *p* < 0.05.

### 3.4 Correlation between hemodynamics and center of pressure

**Figure 4:**
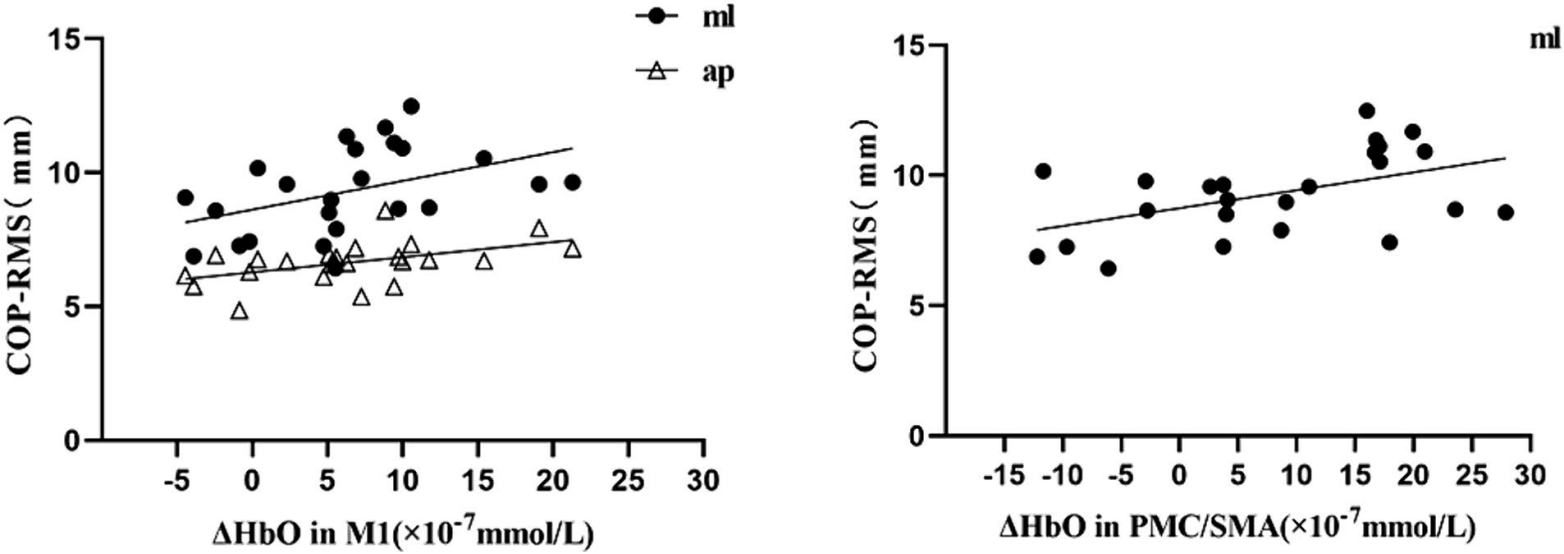
The correlation between △HbO at the channels with significant changes and COP-RMS.

During the single-leg stance task in the CAI group, ΔHbO at Channel 6(M1) had a moderate positive correlation with COP-RMSap (*r* = 0.483, *p* = 0.017) and COP-RMSml (*r* = 0.436; *p* = 0.033) respectively. ΔHbO at Channel 11(PMC/SMA) was moderately positively correlated with COP-RMSml (*r* = 0.488, *p* = 0.016) (see Figure 4).

## 4 Discussion

Our study found that compared to uninjured individuals, the ΔHbO of patients with CAI exhibited a significant increase in the contralateral S1, bilateral M1, and ipsilateral PMC/SMA during single-leg stance with large effect size, respectively. The CAI patients also showed a significant increase in COP-RMS in the mediolateral direction with large effect size, reflecting poorer lateral balance control ability in the ankle joint, with moderate positive correlations with the cortical activation in M1 and PMC/SMA.

Balance ability is fundamental to postural control and a prerequisite for successfully performing daily and sports-related activities (Gebel et al., 2022). CAI is considered a multifaceted disorder with a range of consequences, and the persistent defect in SLS balance control in patients with CAI has been well documented (Doherty et al., 2015). In our study, an increase in COP-RMS in the mediolateral direction in patients with CAI suggested a diminished lateral balance control. Balance control is a complex motor skill that relies on the integration of multimodal information within the sensorimotor cortical network. CAI represents a distinct pathological condition that alters sensorimotor control (Hertel, 2008). It is a widely accepted view that impaired sensorimotor integration within complex neural networks is the cause of balance control deficits in patients with CAI (Hertel, 2008; Pietrosimone et al., 2012; Mohamadi et al., 2020; Suttmiller and McCann, 2020).

The neuroplasticity related to CAI injury is likely attributable to a combination of changes in sensory feedback caused by the injury and compensatory adjustments in behavioral motor control. The destruction of proprioceptors following ankle ligament injury, the ensuing inflammatory cascade, and chronic effusion in the joint may have a prolonged effect on the sensory input of joint position to the CNS, potentially eliciting the maladaptive neuroplasticity that impairs sensorimotor functions (Xue et al., 2022). Under normal circumstances, the sensorimotor loop represents a complex physiological process where incoming information must travel through peripheral mechanoreceptors such as ligaments, ascend along the spinal tracts to reach the somatosensory cortex, and generate a muscular response, which integrates outputs from the motor cortex and spinal reflexes, thereby stabilizing the joint (Needle et al., 2014). Unfortunately, due to afferent neural blockade, ankle injuries may lead to a lack of neural communication between the ligaments and the somatosensory cortex, affecting somatosensory perception in patients with CAI (Zhang et al., 2022), and placing the joint at specific positions that increase the risk of injury (Maricot et al., 2023). Due to the strong connectivity between the primary motor cortex and the somatosensory cortex, peripheral afferent information from somatosensory structures around the ankle joint significantly influences the stability and reorganization of cortical motor output (Kosik et al., 2017).

The M1 is a major contributor to the generation of neural impulses, receiving and processing inputs from almost all cortical areas related to motor control, including the PMC, SMA, and S1, and it sends motor commands via the corticospinal tract to regulate balance control (Chen et al., 2021). The PMC/SMA is involved not only in learning and planning for postural control and balance recovery but also in establishing new motor programs to maintain balance (Li et al., 2023). Previous study found that the SMA activation showed significant variability in CAI patients compared to a healthy control group, indicating that they may alter cortical activation strategies to maintain single-leg balance (Rosen et al., 2019). Stable postural control is associated with cortical activity in the SMA (Kumai et al., 2022). The S1 is responsible for receiving proprioceptive inputs (Xie et al., 2022), including kinesthesia and position sense for the human motor control system. We observed the increased ΔHbO in specific brain regions of CAI patients, reflecting enhanced activation, which suggested that CAI patients may require additional cerebral resources to maintain balance during SLS.

Proprioception and motor control of the distal limbs are primarily attributed to the cortex of the contralateral hemisphere (Lehmann et al., 2021). Our findings also indicate the activation of the contralateral S1 and M1 during SLS in CAI patients. In addition, the ipsilateral PMC/SMA and M1 are activated, which are to decode specific motor intentions (Gao et al., 2022). The SMA has been described as a gateway for interhemispheric motor control, with dense connections between cortical and subcortical motor structures. Through ipsilateral and contralateral projections, SMA influences the control of the contralateral limb via fibers reaching the ipsilateral M1, as well as regulate the effects on the contralateral SMA through callosal connections with it (Ruddy et al., 2016). Furthermore, inter-regional activation (PMC, SMA, and M1) within the motor cortex is quite evident, as they are highly interconnected and receive inputs from sensory pathways for motor control (Khan et al., 2023). In our study, neural activity showed a significant elevation in the ipsilateral M1 and PMC/SMA of CAI patients, consistent with a previous similar study (Ruddy et al., 2017). CAI patients alter their gait and posture to compensate for the injured ankle, implying a reassignment of brain function (Ziabari et al., 2022). Thus, the increased activation of the ipsilateral motor cortex may be a compensatory strategy to maintain overall body balance. When one ankle is injured, the ipsilateral hemisphere may enhance the role of the unaffected ankle in maintaining balance. Pietromone and colleagues discovered that the excitability threshold of the fibularis longus muscle in CAI patients was higher than in healthy individuals, suggesting a reduction in cortical motor excitability of this muscle in CAI patients, thereby affecting their ability to control posture, because the cortical motor excitability of leg muscles is sensitive to changes in postural control and associated with the anticipated postural response (Pietrosimone and Gribble, 2012). This aligns with our findings that CAI patients have poorer balance control in the mediolateral direction, indicating insufficient lateral control over the ankle joint. Additionally, the excitability of the M1 in CAI patients also affects the activation of the sensory cortex; reduced excitability of the M1 need more sensory neural activity to compensate when executing functional movements (Hirtz et al., 2018). The increased cortical activation in the somatosensory regions facilitates the modulation of motor pathways (Sherman et al., 2023), compensating for the sensory afferent blockade caused by trauma and maintaining the performance of motor tasks.

These findings have been corroborated in other musculoskeletal disorders. The patients who have undergone anterior cruciate ligament reconstruction demonstrate a greater reliance on neural sources related to motor preparation and sensory feedback for postural control during the early stage of SLS, which may serve as a compensatory protective mechanism, adapting to altered sensory inputs from the reconstructed knee joint (Neto et al., 2019). In addition, the patients with chronic lower back pain (CLBP) have increased activation in PMC/SMA under different upright postural task conditions, likely indicating that they require more motor resources to maintain balance (Li et al., 2023). When faced with a challenge of balance maintenance, these patients tend to modulate their neural response by increasing the activation of specific brain regions.

## 5 Conclusion

During single-leg stance requiring a high-level balance, the patients with CAI exhibit increased activation in specific brain regions, specifically including the bilateral M1, ipsilateral PMC/SMA, and contralateral S1. This enhanced brain activity may represent a neurophysiological compensatory mechanism for poorer lateral balance control ability in the ankle joint.

## 6 Clinical implications

We observed that CAI patients may rely more on cerebral resources for motor control and sensory feedback to maintain balance. Our research offers a novel perspective in the development of rehabilitation strategies for chronic ankle instability. Firstly, this finding underscores the importance of balance training in rehabilitation strategies, as it may require greater neural system engagement to maintain stability. Secondly, our findings suggest that non-invasive brain stimulation could be a promising treatment approach. By stimulating specific brain regions, we might enhance CAI patients’ neuroplasticity, helping them recover balance function more effectively. This perspective introduces new ideas and possibilities for rehabilitation practices, encouraging clinicians to incorporate balance training and non-invasive brain stimulation into treatment protocols to fully harness the plasticity of the central nervous system. This approach aims to assist patients in functional recovery, reduce the risk of re-injury, and minimize the prevalence of CAI.

## 7 Limitations

This study has certain limitations that must be considered. First, the cross-sectional study design limits our ability to determine the causal relationship between the adaptive cerebral characteristics and the development of CAI. Second, in this study, the static balance assessment employed may not have been sufficiently challenging for the uninjured control group; dynamic assessments, such as single-leg standing on foam or a multi-axial surface, might better reveal differences in balance control, as these are more representative of the demands of everyday dynamic activities. Third, due to the limitations of the functional near-infrared spectroscopy equipment, we were unable to detect activity in other brain regions associated with balance control, such as the prefrontal cortex, cerebellum, midbrain motor areas, and basal ganglia.

## Data availability statement

The raw data supporting the conclusions of this article will be made available by the authors, without undue reservation.

## Ethics statement

The studies involving humans were approved by the Ethics Committee of Shandong Sport University. The studies were conducted in accordance with the local legislation and institutional requirements. The participants provided their written informed consent to participate in this study.

## Author contributions

NL: Data curation, Formal analysis, Investigation, Methodology, Software, Writing – original draft, Writing – review & editing. CY: Data curation, Resources, Software, Visualization, Writing – original draft, Writing – review & editing. QS: Formal analysis, Methodology, Project administration, Resources, Software, Writing – original draft, Writing – review & editing. FY: Data curation, Funding acquisition, Investigation, Project administration, Writing – original draft, Writing – review & editing. YC: Conceptualization, Methodology, Project administration, Supervision, Writing – original draft, Writing – review & editing.

## Supplementary material

Supplementary TABLE 1 MNI coordinates of channels and sources.
