# Supplementary material for: Patients with chronic ankle instability exhibit increased sensorimotor cortex activation and correlation with poorer lateral balance control ability during single-leg stance: a FNIRS study
Source: Front Hum Neurosci. 2024 Apr 26;18:1366443. doi: 10.3389/fnhum.2024.1366443 (PMC11082417; doi:10.3389/fnhum.2024.1366443)
Supplement: Supplementary TABLE 1 — MNI coordinates of channels and sources. [file Table_1.DOCX]

| Channel | MNI coordinates | | |
| --- | --- | --- | --- |
|  | x-axis | y-axis | z-axis |
| CH01 | -23 | -3 | 73 |
| CH02 | -36 | -17 | 72 |
| CH03 | -15 | -16 | 78 |
| CH04 | -26 | -27 | 74 |
| CH05 | -36 | -39 | 71 |
| CH06 | -16 | -41 | 79 |
| CH07 | -27 | -52 | 73 |
| CH08 | -35 | -62 | 64 |
| CH09 | -16 | -61 | 73 |
| CH10 | -26 | -74 | 59 |
| CH11 | 21 | -3 | 74 |
| CH12 | 12 | -18 | 79 |
| CH13 | 34 | -18 | 74 |
| CH14 | 22 | -28 | 77 |
| CH15 | 12 | -41 | 81 |
| CH16 | 33 | -40 | 73 |
| CH17 | 23 | -52 | 75 |
| CH18 | 12 | -63 | 72 |
| CH19 | 34 | -61 | 66 |
| CH20 | 22 | -74 | 62 |

| Source | MNI coordinates | | |
| --- | --- | --- | --- |
|  | x-axis | y-axis | z-axis |
| T1 | -14 | -4 | 76 |
| R1 | -34 | -4 | 67 |
| T2 | 31 | 4 | 67 |
| R2 | 13 | 0 | 75 |
| T3 | -38 | -27 | 71 |
| R3 | -14 | -30 | 79 |
| T4 | 11 | -29 | 80 |
| R4 | 32 | -23 | 74 |
| T5 | -17 | -53 | 75 |
| R5 | -37 | -53 | 69 |
| T6 | 34 | -51 | 71 |
| R6 | 14 | -51 | 78 |
| T7 | -34 | -73 | 56 |
| R7 | -15 | -74 | 64 |
| T8 | 16 | -71 | 67 |
| R8 | 35 | -65 | 62 |
